# Supplementary figures and images for: A novel simple immunoassay for quantification of blood anti-NMDAR1 autoantibodies
Source: PeerJ. 2025 Mar 31;13:e19212. doi: 10.7717/peerj.19212 (PMC11967420; doi:10.7717/peerj.19212)

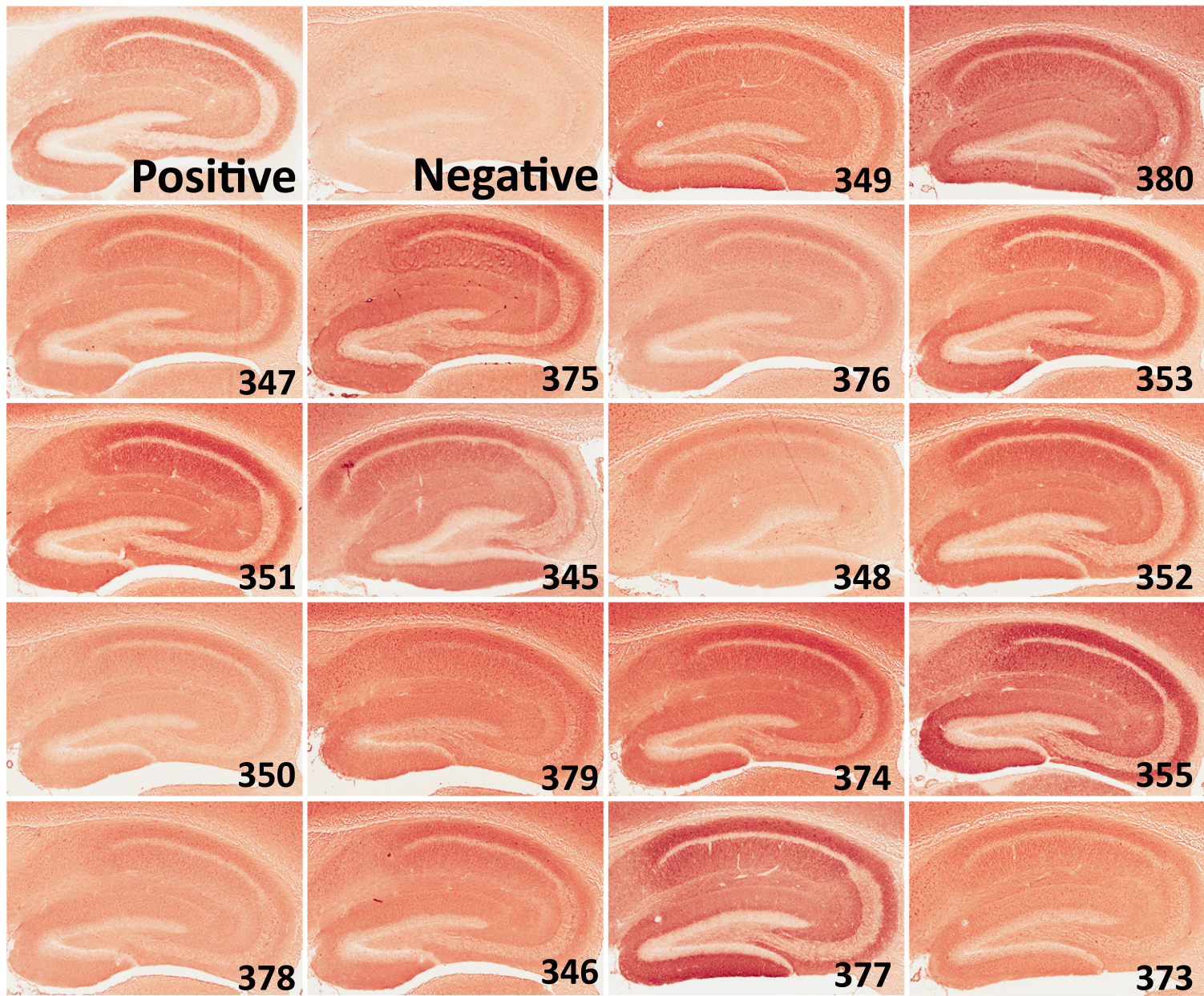

Supplement: Supplemental Information 1 — Mouse ID number was included for each staining. [file peerj-13-19212-s001.pdf]
